# The Silence of the Insurance Commission

**Published:** 1912-04-06

**Authors:** 


### The Hospital

The Workers' Newspaper of Administrative Medicine and Institutional Life, Administration, National Insurance and Health.

No. 1343, Vol. LII. SATURDAY, APRIL 6, 1912. PRICE ONE PENNY.

### THE SILENCE OF THE INSURANCE COMMISSION.

The suffragettes and, more inevitably still, the coal strike have usurped so large a share of public attention and of private recrimination that the issue between the hospitals and the medical profession upon the one hand, and the Insurance Act upon the other, has been almost forgotten. The obscurity thus devolving upon and enveloping the question has not been penetrated by a single ray of light from the Insurance Commissioners. Up to the present no announcements have been made as to whether, and how, the large powers assigned to these officials will be utilised to shape the provision of medical benefit into such form as shall safeguard the voluntary hospitals and the mass of private practitioners. This silence is ominous, we fear, of further disappointments. If the "six-point" policy or anything approaching it is to be granted by the Commissioners, the sooner they make some public intimation of it the better, for July is drawing near, and so far as it is possible to tell, the profession is no nearer obtaining satisfaction than it was six months ago. As for the hospitals, the position is much the same; inasmuch as they cannot vote at parliamentary elections, their position is one degree more hopeless than that of the doctors, whose votes are so few that astute politicians can always afford to disregard them.

The object of this delay is not easy to understand, unless it is thought that the *fait accompli* of the Act in operation will bring the hospitals and the medical profession to heel. All that we have to go upon for enlightenment is sundry speeches, chiefly of the Chancellor's and Mr. Masterman's, breathing threats of dire vengeance upon the doctors if they should dare to hold out for their terms: those terms, be it remembered, which they pressed for whilst the Act was still a Bill, and were told they could negotiate for with the Commissioners. Threats of the suspension of medical benefit are the stock-in-trade of the Chancellor's satellites, and we have already pointed out that such suspension would convert the Act virtually into a dead letter. Probably the object of this bravado is to bluff the profession into accepting something a good deal less than the six-point minimum: to cajole it out of its demand for a living wage, in the jargon of the day.

We believe such tactics will bring their own retribution of failure. In the game of vote-angling the Friendly Societies constitute, it is true, a much finer catch than the despised medical profession, but even here some serious miscalculations seem to have been made, if by-elections are anything to go by. Meanwhile the medical profession is waiting for information and for the result of the negotiations which its representatives may have been conducting with the Insurance Commission. Its solidarity, as far as can be judged, remains unimpaired; though it has still, of course, to be tested by the searching trial of conflict truculently foreshadowed by Mr. Masterman. That solidarity must win the day if only it can be fully maintained; nay, more, it will emancipate the submerged tenth into whose souls the iron of contract practice had entered deeply. Now or never can the battle of the clubs be decided once and for all in favour of the doctors, and most of them seem to realise that now or never must the ranks be locked.

The gist of the whole complicated subject of contract practice has been very admirably and lucidly explained for non-medical readers in a leading article in the *Times* for March 29, and the thanks of all medical men are due to the editor and his contributor for their effort to inform the public exactly how matters stand in regard to contract practice. For medical readers the most noteworthy addition to the literature of the struggle is the issue of a small book * by Mr. E. W. Lowry, M.R.C.S., L.R.C.P., entitled "Can the Doctors Work the Act?" The conclusion reached is that, consistently with their professional honour, the welfare of their patients, and their own personal interests, they cannot do so—unless, that is, very material alterations in the conditions of medical service under the Act are provided by the Insurance Commissioners. Mr. Lowry proceeds to develop the argument for adopting some scheme on lines similar to those of the National Deposit Society, which has done excellent work for many years. He shows that the

* Watts and Co. 1s. net.

216468

system of payment for work actually done is the best and only efficient safeguard against malingering, and in our opinion one of the inevitable results of the haste and carelessness with which the Insurance Act was drafted and passed is the encouragement of malingering, an evil which several other Acts of Parliament have already gone far to create. He shows, too, that the amount of sickness experienced by the National Deposit Society is but a quarter of that of any society working on a capitation basis. The medical service costs less, is better paid, and is more satisfactory both to patients and doctors than that of any other society. On the other hand, the system involves somewhat laborious bookkeeping.

The argument thus briefly summarised is very fully set forth in Mr. Lowry's excellent booklet. At this late stage it is, one may suppose, unlikely that a plan so divergent from that contemplated by the Act will have much chance of favourable consideration. But apart from that the pamphlet still has a real value, because it explains so clearly and emphatically the futile and disastrous policy of perpetuating the present style of friendly society "club doctoring" and exposes the danger to the workers and the injustice to the doctors of the vicious system in whose shackles the profession has for so long been manacled. The discussion of these questions is throughout able and dispassionate; the exposition is lucid and logical. A great deal of excellent common-sense has been packed into a small space, and if the art with which this has been done is such that the artifice is not obvious, the fact merely increases the merit of the achievement. The Insurance Commissioners are apparently in no great hurry to publish the result of their deliberations; let us hope they may find time to read through Mr. Lowry's pregnant pages.

